# Reemergence of Chikungunya Virus in Cambodia

**DOI:** 10.3201/eid1812.120471

**Published:** 2012-12

**Authors:** Veasna Duong, Anne-Claire Andries, Chantha Ngan, Touch Sok, Beat Richner, Nima Asgari-Jirhandeh, Steve Bjorge, Rekol Huy, Sovann Ly, Denis Laurent, Bunheng Hok, Maria Concepcion Roces, Sivuth Ong, Meng Chuor Char, Vincent Deubel, Arnaud Tarantola, Philippe Buchy

**Affiliations:** Author affiliations: Institut Pasteur in Cambodia, Phnom Penh, Cambodia (V. Duong, A.-C. Andries, S. Ong, V. Deubel, A. Tarantola, P. Buchy);; Ministry of Health, Phnom Penh (C. Ngan, T. Sok, R. Huy, S. Ly, M.C. Char,);; Jayavarman VII Hospital, Siem Reap, Cambodia (B. Richner, D. Laurent);; World Health Organization, Phnom Penh (N. Asgari-Jirhandeh, S. Bjorge, M.C. Roces);; Preah Vihear Provincial Health Department, Preah Vihear, Cambodia (B. Hok)

**Keywords:** Chikungunya virus, Cambodia, genome, East Central and South African genotype, Southeast Asia, viruses

## Abstract

Chikungunya virus (CHIKV), probably Asian genotype, was first detected in Cambodia in 1961. Despite no evidence of acute or recent CHIKV infections since 2000, real-time reverse transcription PCR of serum collected in 2011 detected CHIKV, East Central South African genotype. Spatiotemporal patterns and phylogenetic clustering indicate that the virus probably originated in Thailand.

Chikungunya virus (CHIKV; family *Togaviridae*; genus *Alphavirus*) is an arthropod-borne virus transmitted to humans by *Aedes* spp. mosquitoes (*1*). It is an enveloped, positive-sense, single-stranded RNA virus with a genome of ≈11.8 kb (*2*). Three genotypes have been identified: Western African, East Central South African (ECSA), and Asian (*3*).

First identified in Tanzania in the mid-1950s, CHIKV circulated in the 1960s in sub-Saharan Africa and several Asian countries (*4*,*5*). Reemergence of CHIKV (ECSA genotype) was reported in Democratic Republic of Congo in 1999–2000 (*6*,*7*) and in Kenya in 2004 (*4*). This genotype emerged in Comoros followed by Réunion Island, the Seychelles, Mauritius, Mayotte, and India in 2005 (the Indian Ocean outbreak); in Sri Lanka and Malaysia in 2006; in Singapore and Thailand in 2008; and in China in 2010 (*8*).

In Cambodia, CHIKV was first detected in 1961, probably the Asian genotype that was circulating in the region at that time (*9*). Since 2000, all blood specimens collected by the National Dengue Control Program, Ministry of Health Cambodia, during hospital-based surveillance of dengue and investigation of suspected dengue cases have been screened for IgM against CHIKV, dengue virus (DENV), Japanese encephalitis virus (JEV), and other arboviruses. Despite this testing, no evidence of acute or recent CHIKV infections has been found. To confirm CHIKV infection in samples positive by serologic testing, we conducted real-time reverse transcription PCR (RT-PCR) and complete genome sequencing of the samples. In 2011, we detected CHIKV ECSA genotype in patients in Cambodia and analyzed the phylogenetic origin of the strains.

## The Study

**Figure 1:**
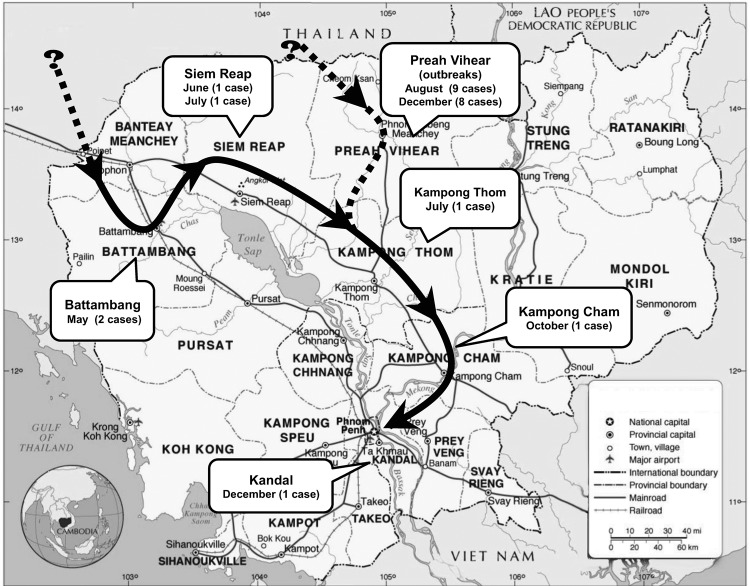
Spatiotemporal pattern of chikungunya cases showing the 6 provinces in which cases occurred and the most likely route of virus introduction into Cambodia, 2011. Adapted, with permission, from www.nationsonline.org/oneworld/map/cambodia-administrative-map.htm.

We obtained samples from 3 sources: national dengue-like surveillance, an encephalitis study, and the outbreak investigation. From surveillance, during 2000–2011, an average of >700 paired serum samples were collected annually from patients admitted to sentinel hospitals for dengue-like syndrome (Battambang, Siem Reap, Kampong Cham, Takeo, Phnom Penh; Figure 1) in the National Dengue Control Program (*10*). From the encephalitis study, conducted July 2010 through July 2011, samples from 196 patients were collected as part of a surveillance study of central nervous system infections in Jayavarman VII hospital in Siem Reap (with written consent from patients or legal guardians and study approval by the National Ethics Committee in Cambodia). During the outbreak investigation, serum was collected during investigations by National Health authorities in Preah Vihear Province on August 16 (n = 9) and December 9–10, 2011 (n = 8), of outbreaks of suspected measles-like or dengue-like illnesses (Table). 

**Table:** Characteristics of 24 patients with positive results for chikungunya virus, Cambodia, 2011*

Patient no.	Project	Age, y/sex	Date of symptom onset	Date sampled	Province	Initial syndrome reported	IgM-capture ELISA			RT-PCR		Full-genome sequencing
							1st	2nd		Serum	SN	
V0603308	NDCP	2/M	May 26	May 28	BTB	Dengue†	Neg	Pos		Pos	Pos	Partial
V0603310	NDCP	4/F	May 26	May 28	BTB	Dengue	Neg	Pos		Pos	Pos	Complete
V0705309	CNS	5/M	Jun 23	Jun 30	SRP	Encephalitis‡	Neg	Pos		Neg	Neg	ND
V0719309	CNS	14/M	Jul 9	Jul 17	SRP	Encephalitis	Pos	Pos		Neg	Neg	ND
V0719310	CNS	7/M	Jul 10	Jul 13	KTH	Encephalitis	Pos	Pos		Pos	Neg	ND
V1005304	NDCP	15/M	Sep 30	Oct 4	KCH	Dengue	Neg	Pos		Pos	Pos	ND
V1024306	CDC	11/F	Aug 10	Aug 16	PVH	Measles§	Neg	NA		Pos	Neg	Complete
V1024307	CDC	11/F	Aug 15	Aug 16	PVH	Measles	Pos	NA		Neg	Neg	ND
V1024308	CDC	28/F	Aug 8	Aug 16	PVH	Measles	Neg	NA		Pos	Neg	Complete
V1024309	CDC	30/F	Aug 8	Aug 16	PVH	Measles	Pos	NA		Pos	Pos	ND
V1024310	CDC	17/M	Aug 13	Aug 16	PVH	Measles	Neg	NA		Pos	Pos	Complete
V1024311	CDC	2.5/F	Aug 16	Aug 16	PVH	Measles	Neg	NA		Pos	Pos	Complete
V1024312	CDC	13/F	Aug 16	Aug 16	PVH	Measles	Neg	NA		Pos	Pos	ND
V1024313	CDC	10/F	Aug 14	Aug 16	PVH	Measles	Neg	NA		Pos	Pos	Complete
V1024314	CDC	31/F	Aug 15	Aug 16	PVH	Measles	Neg	NA		Pos	Pos	Complete
V1214306	NMC	49/M	Dec 9	Dec 9	PVH	Dengue	Neg	NA		Pos	Neg	ND
V1214307	NMC	56/M	Dec 6	Dec 9	PVH	Dengue	Neg	NA		Pos	Neg	ND
V1214308	NMC	29/M	Dec 5	Dec 9	PVH	Dengue	Neg	NA		Pos	Pos	ND
V1214309	NMC	39/F	Dec 9	Dec 10	PVH	Dengue	Neg	NA		Pos	Pos	ND
V1214310	NMC	17/F	Dec 9	Dec 10	PVH	Dengue	Neg	NA		Pos	Pos	ND
V1214311	NMC	27/M	Dec 7	Dec 10	PVH	Dengue	Pos	NA		Neg	Neg	ND
V1214312	NMC	16/M	Dec 9	Dec 10	PVH	Dengue	Neg	NA		Pos	Pos	ND
V1214333	NMC	30/F	NA	Dec 10	PVH	Dengue	Neg	NA		Pos	Pos	ND
V1207304	NDCP	9/F	Dec 1	Dec 6	KAN	Dengue	Pos	Pos		Neg	Neg	ND

*All patients had negative IgM-capture ELISA results for flaviviruses (dengue 1–4, Japanese encephalitis, Langat) and other alphaviruses (Sindbis).  RT-PCR, real-time reverse transcription PCR; 1st, acute-phase serum sample; 2nd, convalescent-phase serum sample; SN, supernatant; NDCP: National Dengue Control Program, National Malaria Center, Ministry of Health; BTB, Battambang; Neg, negative; Pos, positive; CNS, central nervous system infection (encephalitis) study; SRP: Siem Reap; ND, not done; KTH: Kampong Thom; KCH: Kampong Cham; CDC, Communicable Disease Control Department, Ministry of Health, Preah Vihear outbreak investigation in August 2011; PVH: Preah Vihear; NA, not available; NMC, National Malaria Center, Ministry of Health, Preah Vihear outbreak investigation in December 2011; KAN, Kandal. †Dengue-like: fever with headache, retro-orbital pain, myalgia, joint pain, and/or hemorrhage. ‡Encephalitis: fever with convulsion, vomiting, and/or hemiplegia. §Measle-like: fever with rash, cough, or join pain.

All serum was tested at the Institut Pasteur in Cambodia. Acute-phase and/or convalescent-phase specimens were tested for IgM against flaviviruses (DENV, JEV, and Langat virus) and alphaviruses (CHIKV Ross C 347 strain and Sindbis virus). We used in-house IgM-capture ELISA as described by Vong et al. (*11*) with JEV, Langat, Sindbis, DENV, and CHIKV antigens; CHIKV was isolated by use of a mosquito cell line (clone C6/36 of *Aedes albopictus* cells) (*11*). Viral RNA was extracted from 140 µL of serum by using the QIAamp Viral RNA Mini Kit (QIAGEN, Hilden, Germany) according to manufacturer’s recommendations. The presence of CHIKV RNA was determined by real-time RT-PCR selective for the *E1* gene, according to a protocol adapted from Pastorino et al. (*12*) for a different Taq polymerase kit (SuperScript III Platinum One-Step Quantitative RT-PCR kit; Invitrogen, Carlsbad, CA, USA). Each series of tests included a negative control.

Among 19 samples positive for CHIKV by real-time RT-PCR, 8 were selected for complete genome sequencing. A total of 22 overlapped PCR products were obtained by using primers published by Schuffenecker et al. (*13*) and sent to Macrogen (Seoul, South Korea) for sequencing.

Sequence assembly and alignment were performed by using the CLC Main Workbench 5.5 package (CLC bio A/S, Aarhus, Denmark). The complete coding region (11,319 nt) of 8 CHIKV isolates from Cambodia were aligned with 64 reference strains available in GenBank. Phylogenetic analysis was performed by using the maximum-likelihood approach incorporating the GTR+Γ4 model of nucleotide substitution with a bootstrap resampling process of 1,000 replications.

During this reemergence of CHIKV in 2011, a total of 24 patients with fever, sometimes associated with acute arthritis or encephalitis (suggesting that many classical infections were not reported because encephalitis is a rare complication of chikungunya), had positive RT-PCR and/or IgM-capture ELISA results for CHIKV. The first 2 cases were identified in May 2011 by the National Dengue Control Program in Battambang Province (eastern Cambodia) near the Thailand border (Figure 1). These 2 cases were in children hospitalized for suspected dengue. Subsequent cases were reported the same year in Siem Reap (2 cases, June and July), Kampong Thom (1 case, July), Kampong Cham (1 case, October), and Kandal (1 case, December) Provinces. Two other outbreaks were documented in villages in Preah Vihear Province (northern Cambodia) in August (9 cases) and December (8 cases) 2011 (Table). The sequence of outbreaks, in time and space, suggests that the virus was introduced to areas bordering Thailand and progressed through Cambodia, affecting city and villages along major northwest to southeast routes (Figure 1). Average patient age was 20 years (range 2–56 years); cases were distributed equally among male and female patients.

## Conclusions

**Figure 2:**
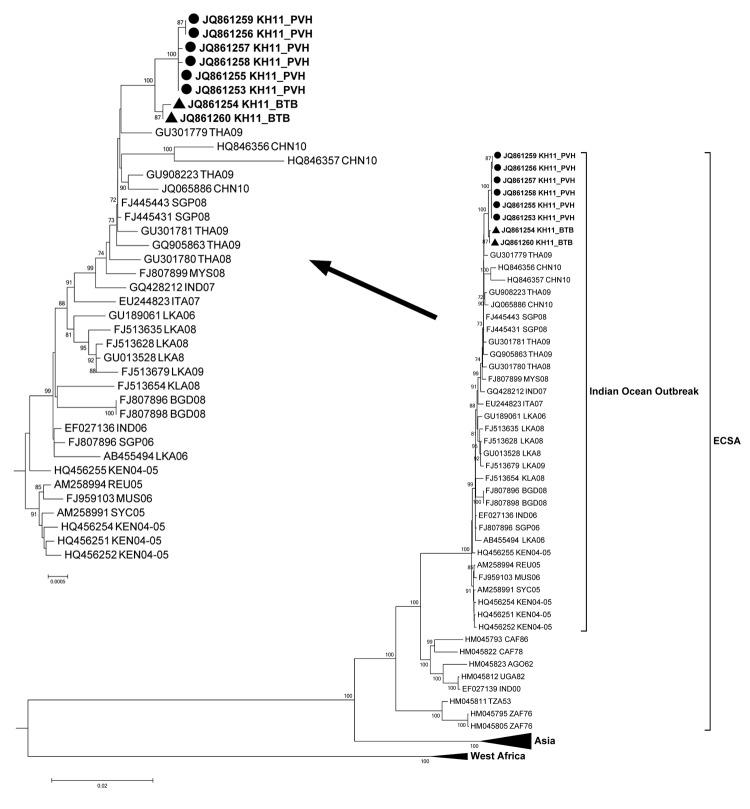
Phylogenetic tree based on the whole genome of chikungunya virus (CHIKV). Viruses were identified by using the GenBank accession number, country code, and year of isolation. Boldface indicates strains from Cambodia; circles indicate isolates from Preah Vihear Province; triangles indicate strains from Battambang Province. Arrow indicates enlarged Indian Ocean outbreak strains. All 8 strains from Cambodia carried the A226V mutation. Numbers represent the bootstrap support obtained for respective branches (>70). The tree was rooted by o’nyong-nyong virus (GenBank accession no. AF079456, UGA96-ONNV). ECSA, East Central South African genotype. Scale bars indicate nucleotide substitutions per site.

The alignment of the *E1* gene sequence of CHIKV showed that all 8 strains carried the same amino acid substitution in the E1 protein (E1-A226V) as did the strains that were naturally selected by the mosquito vector a few months after the beginning of the Indian Ocean outbreak (*13*). Phylogenetic analysis of the complete genome sequence revealed that all viruses from Cambodia clustered with those isolated during the Indian Ocean outbreak and within the ECSA phylogenetic group (Figure 2). These isolates from Cambodia were closely related to the viruses isolated from southern Thailand during the 2008–2009 outbreak with bootstrap values <70 (data not shown) and to other isolates from the recent outbreaks in Asia (Singapore, Malaysia, Indonesia, and China). The pairwise nucleotide comparison of the complete coding region showed a high average percentage of similarity (>99.50%) with the recent isolates from Thailand, Malaysia, Singapore, and China. The identity between the strains from Cambodia ranged from 99.89% to 99.93% at the nucleotide level. Of note, the Cambodian strains can be separated into 2 groups supported by a bootstrap value of 100, suggesting that the viruses isolated in Battambang and Preah Vihea Provinces, which each border Thailand, could have been introduced separately, although we cannot exclude the possibility of introduction from other Asian countries as well.

As numbers of reported cases, numbers of provinces affected, and abundance of mosquito vectors (*Ae. aegypti* and *Ae. albopictus*) increase, the risk for local transmission will probably increase in the next few years, and levels of CHIKV infection could reach those of DENV infection. The ECSA genotype could then become endemic to Cambodia, which could face the same situation as in the 1960s, when a number of chikungunya cases were reported in Cambodia, although the 1960s epidemic did not last long (according to data available) and was not followed by continuous virus circulation leading to successive outbreaks. As CHIKV reemerges after 50 years of absence or low-level transmission, cocirculation with DENV might cause substantial challenges for public health, especially hospital overloading and increased needs for case management. This outbreak of CHIKV ECSA genotype spread rapidly in Cambodia over a short 7-month period. The outbreak should serve as a warning for health authorities to prepare, not only in Cambodia, but also in other areas where, to our knowledge, this genotype has not been reported, such as Vietnam and Lao People’s Democratic Republic. 
